# Abbreviations

**Published:** 1902-10-18

**Authors:** 


### ABBREVIATIONS.

A little while ago a nurse, observing a strange diagnosis, asked us the meaning thereof. The mystic letters were "G. O. K.", and the nurse, being, we suppose, of a curious turn of mind, inquired what disease they indicated, what was the treatment of the malady, whether there was any cure for it, and whether it was likely to be a long case? Such laudable curiosity was not long unsatisfied, for another nurse soon supplied the information that the letters mentioned were but an abbreviation of the expression "God only knows." This reminds us of the somewhat analogous expression "N. Y. D.", which is said to figure in the returns of certain military hospitals, the meaning of this cryptic saying being that the case is "Not yet diagnosed." All of which is very ridiculous, and serves well to show the absurdity of that most objectionable form of slang which consists of the use of arbitrary abbreviations which, without their context, are often absolutely meaningless. So deeply rooted has this bad habit become among medical men that a recently published medical dictionary ("The American Illustrated Medical Dictionary," W. B. Saunders and Co.) finds it worth while to devote several pages to definitions of these abbreviations, and a very cursory survey of these pages suffices to show the errors into which a person might easily be led by such a misuse of letters. In many cases indeed their interpretation depends entirely upon the context. "A. D." means, we are told, either "Anodal duration" or "*auris dextra*, right ear." "As." means "arsenic," but it also means "astigmatism." "C." has quite a repertoire of different meanings, such as "carbon," "cathode," "centigrade," "clonus," "closure," "color sense," "*congius*, gallon," and "cubic." "Cc." means "cathodal closure" and "cubic centimeter." "D." stands according to the context for either "*da*, give," "dexter," "diopter," "*dosis*, dose" or "duration." "F." again is another letter with many meanings. It may mean "Fahrenheit," or "*fiat*, let there be made," or "field of vision," or "fluorin," or "formula." "K." we are told means either "electro-static capacity," "kathode," or "potassium," while "S." may either be "*semis*, half," "sight," "sign," "south pole," or "sulphur." Evidently the use of abbreviations having such very indefinite meanings is a practice to be sedulously avoided. Who is to guess that "Z.Z.' Z.''" means "increasing degrees of contraction," or that "E.-j." means "elbow-jerk," or that while "P.D." means "potential difference," "PD." means "prism-diopter," and "Pd." means "palladium." Again, "H. D. L. W." is very significant when one knows what it means, but until one has learned the language one might hardly guess that it indicates the "distance at which a watch is heard by the left ear." But it is when we come to the contractions used for Latin words and phrases that the full oddity of this slang tongue betrays itself. "Ad def. an." is given as meaning "to fainting," which when interpreted by way of "*ad defectionem animi*" is very clear. What, however, are we to say to "Ad 2 vic." which is interpreted "*ad duas vices*, at two times, for two doses." "C. m. s." which our clerical friends interpret "church missionary society," is here given as meaning "to be taken to-morrow morning." "Detur in 2plo" is an Americanised contraction for "*detur in duplo*," or "let twice as much be given." "H.p.n." is made to stand for "*haustus purgans noster*, our own purgative draught," surely a relic of the time when each country apothecary had a pride in his own particular brand of aperient. There is much to be said against the custom of prescribing in Latin, but if we do persist in airing our classics (!) at least let us write in full so that by aid of a dictionary the dispenser may be able to extract some meaning from our prescriptions, and do not let us befog all accuracy of thought and intention in a cloud of clumsy abbreviations. Yet there is one which, if it is to be used, may perhaps be properly cut down to the most unintelligible proportions, namely, "Ne. tr. s. num." which the dispenser's boy is supposed to interpret as follows: "*ne tradas sine nummo*, do not deliver unless paid!" But surely the acme of absurdity in the use of contractions is reached when the surgeon, on being asked what is the prognosis in his case, solemnly answers "G.O.K."

